# International Regulatory Classification of Minimally Manipulated and Borderline Cell and Tissue Products: Implications for Mesenchymal Stromal Cell-Based Interventions

**DOI:** 10.3390/bioengineering13101235

**Published:** 2026-10-21

**Authors:** Airat Bilyalov, Nikita Filatov, Natalia Iashina, Andrey Briko, Pavel Chibizov, Irina Nadelyaeva, Alexey Tikhomirov, Anastasia Petrikina, Viatcheslav Vasilyev, Roman Deev, Ilya Eremin, Konstantin Kotenko

**Affiliations:** 1 Petrovsky National Research Centre of Surgery, Moscow 117418, Russia; 2 Kazan State Medical Academy, Branch Campus of the Russian Medical Academy of Continuous Professional Education, Ministry of Health of the Russian Federation, Kazan 420012, Russia

**Keywords:** regenerative medicine, minimally manipulated products, cell therapy, mesenchymal stromal cells, stromal vascular fraction, advanced therapy medicinal products, regulatory framework, homologous use, substantial manipulation, biomedical cell products

## Abstract

Cell and tissue products used in regenerative medicine are classified differently across jurisdictions according to processing, intended use, and manufacturing setting. This review compares regulatory approaches to products including bone marrow aspirate concentrate, adipose-derived stromal vascular fraction (SVF), microfragmented adipose tissue, nanofat, amniotic products, and cartilage-derived products, with particular attention to their implications for mesenchymal stromal cell (MSC)-based interventions. Legislation, regulatory guidance, enforcement documents, judicial decisions, and scientific publications were examined for the United States, European Union, Russian Federation, Eurasian Economic Union, selected Asian countries, and Arab jurisdictions. In the United States, minimal manipulation and homologous use are central classification criteria. In the European Union, substantial manipulation or use for a different essential function may bring a product within the advanced therapy medicinal product framework. Russian and Eurasian rules require distinction among biomedical cell products, individualized products, medicinal products, transplantation materials, and medical procedures. SVF is a heterogeneous cell population, while microfragmented adipose tissue retains tissue structures. Neither should be equated with an isolated or culture-expanded MSC preparation solely because it contains stromal cells. Regulatory assessment and interpretation of clinical evidence require clear reporting of product composition, processing, claimed mechanism of action, and manufacturing conditions.

## 1. Introduction

This review focuses on uncultured cell and tissue preparations, including bone marrow aspirate concentrate, adipose tissue derivatives, amniotic products, and cartilage-derived preparations. “Minimally manipulated” is used as a working descriptive label for the preparations examined here, without implying that each satisfies a regulatory threshold for processing. Culture-expanded cell products are discussed as comparators where relevant to classification and clinical evidence.

In regulatory discussions, “minimal manipulation” refers to the criterion established within the specified jurisdiction, particularly the US HCT/P framework. “Minimally processed” is used descriptively unless a jurisdiction-specific definition is stated. The EU concept of “substantial manipulation” has its own statutory meaning and is not treated as the direct inverse of the US criterion [1,2,3,4]. “Borderline” describes preparations whose characteristics and intended use require careful assessment to determine the applicable framework for tissue transplantation, procedural use, or cell-based medicinal products. It does not denote a shared legal category across jurisdictions.

Limited processing alone does not establish eligibility for a regulatory exemption or a less extensive authorization pathway. The applicable criteria and their legal consequences differ among jurisdictions [1,2,3,4].

Different jurisdictions apply different regulatory mechanisms to classify these products. In the United States, classification is based on minimal manipulation and homologous use within the regulatory framework for human cells, tissues, and cellular and tissue-based products [2]. In the European Union, the assessment is based on substantial manipulation and on whether the cells or tissues perform the same essential function in the recipient as in the donor [3,4]. In the Russian Federation, the 2023 and 2024 amendments changed the regulatory context for biomedical cell products and introduced a special regime for individualized biomedical cell products manufactured for a specific patient within a medical organization [5,6].

Previous studies have compared regulatory frameworks for regenerative medicine and advanced therapy medicinal products, particularly in the United States and the European Union [7,8]. The classification of borderline minimally manipulated products in relation to recent enforcement practice, judicial decisions, and legislative reforms across a broader set of jurisdictions remains less systematically addressed. This review links the applicable regulatory frameworks to recent enforcement practice, using judicial decisions, regulatory warning letters, and legislative reforms from 2023 to 2025 as examples of how classification criteria are applied in practice. An additional outcome of this work is a set of requirements for describing such products in scientific publications and clinical protocols, formulated on the basis of comparative analysis and applicable regardless of the legal system in which the study is conducted.

The aim of this review is to provide a comparative analysis of the legal status of minimally manipulated cell and tissue products in contemporary regenerative medicine. The review examines approaches used in the United States, the European Union, the Russian Federation, and the Eurasian Economic Union, as well as selected regulatory models in Asian countries and the Arab region. Particular attention is given to how different systems distinguish between tissue transplantation, a medical procedure, a product manufactured for an individual patient, a biological medicinal product, and an advanced therapy medicinal product.

We also examine how regulatory criteria apply to heterogeneous cell and tissue preparations that may contain mesenchymal stromal cells (MSCs), and how these preparations differ from isolated or culture-expanded MSC products. Regulatory classification does not itself demonstrate clinical efficacy, and the presence of stromal cells does not establish that these cells mediate a therapeutic effect [9,10,11].

Computational methods can assist therapeutic candidate selection by predicting associations that can subsequently be investigated experimentally. For example, Tang et al. applied an indicator regularized non-negative matrix factorization method to prioritize potential drug candidates for COVID-19 [12]. For cell and tissue interventions, the assessment of a proposed therapeutic application additionally requires characterization of the administered preparation and experimental and clinical evidence relevant to its intended use [9,11].

## 2. Review Methodology and Criteria for Comparative Analysis

This review was conducted as a narrative comparative legal analysis of regulatory approaches to minimally manipulated cell and tissue products. The analysis focused on the criteria used by different jurisdictions to determine whether a product should be treated as a tissue product, a medical procedure, an individually manufactured product, a biological medicinal product, a biomedical cell product, or an advanced therapy medicinal product.

Scientific literature was identified through PubMed and Google Scholar. Legal and regulatory materials were retrieved from official repositories and competent-authority websites, including the Electronic Code of Federal Regulations, EUR-Lex, the Official Internet Portal of Legal Information of the Russian Federation, and the EAEU legal portal. Source selection was narrative and guided by relevance to product classification, processing, intended use, and the comparative questions addressed in this review. The initial search and source verification were completed on 22 June 2026. Selected regulatory and clinical sources were additionally checked during manuscript revision, with the relevant access dates reported in the reference list. The main sources included documents from the FDA, EMA, EUR-Lex, official legal portals of the Russian Federation, the Eurasian Economic Commission, PMDA, HSA Singapore, MFDS, SFDA, Dubai Health Authority, and the Department of Health Abu Dhabi. Scientific publications were used to support the description of product types, technological characteristics, clinical use, standardization issues, and previously published regulatory comparisons.

Search terms included combinations of the following keywords: “minimally manipulated”, “minimally processed”, “cell and tissue products”, “human cells, tissues, and cellular and tissue-based products”, “HCT/P”, “advanced therapy medicinal products”, “ATMP”, “biomedical cell products”, “stromal vascular fraction”, “bone marrow aspirate concentrate”, “microfragmented adipose tissue”, “nanofat”, “amniotic products”, “cartilage cell products”, “homologous use”, “substantial manipulation”, “hospital exemption”, “same surgical procedure exception”, “regenerative medicine regulation”, “cell therapy regulation”, “tissue engineering regulation”, “mesenchymal stromal cells”, “MSC”, “adipose-derived stromal cells”, and “adipose-derived stem cells”. Searches were conducted in English and Russian. Official regulatory documents in other languages were used when English translations or authoritative summaries were available.

Legislation and regulations provided the basis for describing legal requirements. Regulatory guidance, official explanations, enforcement documents, and judicial decisions were considered according to their respective legal status and scope. Secondary literature supported biological characterization and clinical or regulatory context but was not used to override applicable legislation or replace primary regulatory sources. Scientific articles and reviews were included when they addressed product characterization, clinical use, manufacturing variability, reporting standards, or comparative regulatory analysis. Sources were excluded if they were opinion pieces without regulatory or scientific documentation, commercial materials without verifiable regulatory relevance, or general discussions of cell therapy without product-specific clinical, manufacturing, or regulatory evidence. Publications addressing MSC identity criteria, nomenclature, and the characterization of uncultured adipose- and bone marrow-derived preparations were additionally included to support the distinction between heterogeneous cell and tissue preparations and defined MSC products.

Regulatory provisions were interpreted using the version effective at the stated verification date. Earlier versions were retained only where needed to explain a legislative change or a historical decision and were identified accordingly. Publication and entry into force were distinguished when these dates differed.

Jurisdictions were purposively selected to illustrate contrasting approaches to product classification and clinical-use pathways, rather than to provide an exhaustive global regulatory survey. The United States and the European Union were included because they provide explicit regulatory criteria for minimal manipulation, homologous use, substantial manipulation, and advanced therapy medicinal products. The Russian Federation and the Eurasian Economic Union were included because legislative changes have affected the distinction between biomedical cell products, individualized biomedical cell products, and high-technology medicinal products. The latter category encompasses gene therapy, somatic cell therapy, and tissue-engineered medicinal products under the EAEU framework [5,6,13]. Japan, China, South Korea, and Singapore were selected as examples of Asian regulatory models that combine product-based regulation, institutional clinical-use pathways, conditional or risk-based approaches, and administrative oversight. Saudi Arabia and the United Arab Emirates were included as selected Arab jurisdictions in which regulatory frameworks for stem cell and regenerative medicine technologies are becoming more formalized. The Asian and Arab jurisdictions are illustrative examples. Their inclusion does not imply comprehensive regional coverage or that they represent all regulatory approaches within those regions.

The comparative analysis was performed across several parameters: legal category of the product, source material, autologous or allogeneic origin, degree of processing, preservation of structural or biological properties, homologous use, substantial manipulation, place of manufacture, individualized or serial nature of production, claimed mechanism of action, quality and safety requirements, adverse event reporting, and post-application follow-up. Particular attention was given to borderline products whose classification depends on both biological characteristics and clinical claims, including bone marrow aspirate concentrate, stromal vascular fraction, microfragmented adipose tissue, nanofat, amniotic products, and cartilage-derived products. During revision, targeted supplementary searches identified clinical studies and regulatory assessments relevant to the interpretation of MSC-based interventions. Examples were selected to illustrate product authorization, reassessment following confirmatory evidence, and controlled evaluation of heterogeneous preparations. Study design, sample size, comparator, endpoint definition, assessment time, numerical outcomes, and safety findings were extracted from original publications and regulatory documents. Results were presented using the analysis populations and effect estimates reported by each source. This selection was intended to support the comparative discussion and did not constitute a systematic review or meta-analysis of MSC efficacy.

No lower publication-year restriction was applied. Sources were selected narratively according to their relevance to the comparative questions addressed. Searches and source assessment were undertaken collaboratively, without a formal independent duplicate-screening procedure. Differences in regulatory interpretation were discussed by the authors with reference to the applicable primary legal texts and official guidance. A complete retrieval and screening log was not maintained, so the initial number of records cannot be reliably reconstructed. The final reference list comprises 79 sources, including scientific publications and legal and regulatory documents.

## 3. Regulatory Approaches to Minimally Manipulated Cell and Tissue Products

Regulatory approaches to cell and tissue products differ in their legal categories and conditions for clinical use. The following subsections compare the jurisdictions according to the applicable framework, the criteria used to assess processing and intended use, and the distinction between product authorization and use within a medical institution. Table 1 identifies the competent authorities and summarizes their responsibilities alongside the regulatory frameworks and classification criteria. The comparison distinguishes scientific assessment, marketing authorization, and oversight of clinical activities within medical institutions [1,2,3,4,14,15].

### 3.1. United States

In the United States, the main regulatory source is 21 CFR Part 1271. This part regulates human cells, tissues, and cellular and tissue-based products. A less stringent regulatory regime may apply only when several conditions are met simultaneously. These include minimal manipulation, homologous use, restrictions on combination with other components, and additional criteria related to the systemic effect of the product and the metabolic activity of living cells [1,2]. Regulation solely under Section 361 and Section 21 CFR Part 1271 requires compliance with all criteria in 21 CFR 1271.10(a). This differs from exclusion from the HCT/P definition under 21 CFR 1271.3(d) and from an establishment exception under 21 CFR 1271.15. An HCT/P that meets neither the Section 361 criteria nor an applicable exception is regulated as a drug, device, and/or biological product under 21 CFR 1271.20 [2]. A specific feature of the US approach is the distinction between structural and nonstructural tissues. Structural tissues are tissues whose main function is related to support, protection, volume replacement, mechanical strength, or barrier function. For such tissues, regulatory assessment includes cells, extracellular matrix, spatial organization, and mechanical properties. Nonstructural tissues include cells and tissues whose main function is determined primarily by biological activity, including growth factor secretion, metabolic functions, and immune effects. Accordingly, for structural tissues, regulators assess the preservation of characteristics relevant to reconstruction, repair, or replacement. For cells and nonstructural tissues, they assess the preservation of relevant biological characteristics [1,2]. Therefore, the same processing method may have different legal significance depending on which function of the material is considered primary.

### 3.2. European Union

In the European Union, cell and tissue preparations are assessed against the statutory definitions of the relevant product categories. For somatic cell therapy and tissue-engineered products, substantial manipulation and use for a different essential function are alternative criteria concerning the cells or tissues. Classification also depends on the product’s intended therapeutic action and the remaining elements of the applicable definition. Substantial manipulation should therefore not be presented as a stand-alone rule that automatically classifies every preparation as an ATMP [3,16,17]. For example, using adipose tissue to restore adipose tissue differs functionally from using adipose-derived cells to regenerate articular cartilage. The latter application requires assessment of a different intended function, even if processing is limited. This illustrates the functional criterion rather than establishing the classification of a particular preparation [3,16].

The EU hospital exemption is implemented nationally under Article 3(7) of Directive 2001/83/EC. It concerns ATMPs prepared non-routinely to specified quality standards for an individual prescription and patient, and used in a hospital within the same Member State under a medical practitioner’s exclusive professional responsibility. Manufacturing requires authorization by the national competent authority. National quality, traceability, and pharmacovigilance requirements must be equivalent to the relevant EU requirements. This pathway is distinct from the US same-surgical-procedure exception [2,3,17,18].

### 3.3. Russian Federation and Eurasian Economic Union

In the Russian Federation, cell products are regulated primarily by Federal Law No. 180-FZ “On Biomedical Cell Products”. After the adoption of Federal Law No. 466-FZ, this regulatory framework was substantially amended [5]. The mandatory link between a cell line and ex vivo cell culture was removed from the definition of a cell line, and provisions were introduced to distinguish biomedical cell products from high-technology medicinal products regulated under the rules of the Eurasian Economic Union [5,19]. This affects the assessment of minimally processed products because the absence of culture expansion is not sufficient to exclude a product from the biomedical cell product framework [5]. An additional feature of the Russian model is the regime for the use of individualized biomedical cell products. Decree of the Government of the Russian Federation No. 384 established a procedure for the circulation of products intended to fulfil an individual medical prescription and specifically manufactured for an individual patient directly within the medical organization where they are used [6]. This regime has its own administrative logic and does not correspond either to the US exception for the use of material within the same surgical procedure or to the European hospital exemption for advanced therapy medicinal products [2,3,6]. Its regulatory function is to create a special pathway for individualized use of a cell product while maintaining authorization control, expert assessment, quality control, and reporting requirements [6].

The Russian biomedical cell-product framework and EAEU medicinal-product rules have distinct scopes. Federal Law No. 180-FZ excludes transplantation materials and specified high-technology medicinal products subject to registration under EAEU law and/or Russian medicinal-product legislation from its biomedical cell-product definition [5,19]. EAEU Decisions No. 78 and No. 89 govern registration and evaluation of medicinal products and studies of biological medicinal products, respectively [13,20]. Assessment therefore requires identifying the applicable product category before determining the authorization requirements.

### 3.4. Japan

Japan was among the first countries to establish regenerative medical products as a separate legal category and, at the same time, to introduce a special procedure for the clinical use of the corresponding medical technologies [21,22,23]. This distinction allows market authorization of a product and clinical use of a medical technology to be regulated separately.

### 3.5. China

In China, cell therapy is regulated through two main pathways [24,25]. Some cell products are treated as medicinal products under the system of the National Medical Products Administration. In parallel, certain cell technologies may be used in medical institutions provided that requirements for clinical research and administrative control are met [14,25,26,27]. The Regulations on the Administration of Clinical Research and Clinical Application of New Biomedical Technologies, effective from 1 May 2026, further distinguish clinical research from subsequent clinical application. Technologies within their scope require academic and ethical review and research filing, while clinical application following research requires separate approval. Clinical trials conducted to develop medicinal products or medical devices remain governed by the corresponding legislation [28].

### 3.6. South Korea

In South Korea, regulation is based on the Act on the Safety of and Support for Advanced Regenerative Medicine and Advanced Biological Products [29,30]. The Act distinguishes advanced regenerative medicine as a medical intervention involving cell therapy, gene therapy, and tissue engineering from advanced biological products as medicinal products, including cell-based, gene therapy, and tissue-engineered products. At the same time, certain interventions based on simple processing of cells or tissues are excluded from the scope of advanced regenerative medicine if their biological properties are preserved [29,31,32].

### 3.7. Singapore

In Singapore, cell, tissue, and gene therapy products are regulated under the Health Products Act and the Health Products Cell, Tissue and Gene Therapy Products Regulations 2021. The Health Sciences Authority system is based on risk-based classification [33,34]. Class 1 products are considered lower-risk products only when several conditions are met simultaneously: minimal manipulation, homologous use, and no combination with therapeutic products or medical devices. Other cell, tissue, and gene therapy products are assigned to Class 2 and are subject to more stringent regulation.

Cells and tissues that are minimally processed and reimplanted into the same patient for homologous use within the same surgical procedure are separately excluded from the regime for cell, tissue, and gene therapy products. Minimally processed bone marrow, peripheral blood, umbilical cord blood, or placental blood used for homologous purposes are also excluded [15,33,34].

### 3.8. Saudi Arabia

In Saudi Arabia, the Saudi Food and Drug Authority has published guidance on the classification of advanced therapy medicinal products, including gene therapy, cell-based, tissue-engineered, and combined products [35]. This document indicates a shift toward a more formalized assessment system close to the international approach to advanced therapy medicinal products. It takes into account the degree of cell and tissue processing, the presumed mechanism of action, and the presence of additional components in the product [35].

### 3.9. United Arab Emirates

At the federal level, the Emirates Drug Establishment (EDE) is responsible for medical product classification and registration within its remit. This role should be distinguished from the regulation of healthcare facilities and clinical services by the competent health authorities in individual emirates [36]. In the United Arab Emirates, regulation of stem cells and regenerative medicine is implemented at both the federal level and the level of individual emirates. The Ministry of Health and Prevention, the Department of Health Abu Dhabi, and the Dubai Health Authority are involved in this area [37,38]. This system is characterized by requirements for licensing medical organizations, ensuring the quality and safety of cell products, ethical oversight, and the clinical implementation of cell technologies. Differences in emirate-level requirements make it necessary to identify both the competent authority and the applicable requirements for the product and the clinical service [36,37,38].

**Table 1:** Comparison of regulatory frameworks and competent authorities for cell and tissue products across the selected jurisdictions.

Jurisdiction	Competent Authorities and Principal Responsibilities	Main Regulatory Framework	Principal Considerations for Classification	Distinctions Relevant to Clinical Use
United States	Food and Drug Administration (FDA), particularly the Center for Biologics Evaluation and Research (CBER), oversees cellular therapies and human tissue products within its remit [39].	Human cells, tissues, and cellular and tissue-based products under 21 CFR Part 1271.	Minimal manipulation and homologous use, together with restrictions on combination and criteria concerning systemic effects and the metabolic activity of living cells.	Regulation solely under Section 361 requires all applicable criteria to be met. The same surgical procedure exception is assessed separately.
European Union	European Medicines Agency (EMA), including the Committee for Advanced Therapies (CAT) and the Committee for Medicinal Products for Human Use (CHMP), conducts scientific assessment. The European Commission grants centralized marketing authorizations. National competent authorities oversee the hospital exemption and other nationally administered requirements [3,17,40].	Advanced therapy medicinal products and legislation governing tissues and cells.	Substantial manipulation or use for a different essential function, considered together with the product’s intended action.	Centralized ATMP marketing authorization and the national hospital exemption are distinct pathways. The hospital exemption retains requirements for manufacturing authorization, quality, traceability, and pharmacovigilance.
Russian Federation	Ministry of Health oversees biomedical cell product registration and clinical trial authorization and grants permissions under the individualized product regime. The Federal Service for Surveillance in Healthcare (Roszdravnadzor) licenses biomedical cell product manufacturing and performs regulatory supervision [5,6,19,41].	Biomedical cell products under Federal Law No. 180-FZ, as amended, and rules for individualized biomedical cell products.	Product composition, intended use, and manufacturing conditions. Absence of culture expansion alone does not determine exclusion from the biomedical cell product framework.	Individualized biomedical cell products are subject to a specific authorization regime for manufacture and use within the treating medical organization.
Eurasian Economic Union	Eurasian Economic Commission (EEC) establishes common regulatory rules. Competent authorities of the Member States perform medicinal product assessment and registration under the applicable Union procedures.	Common rules for medicinal product registration and evaluation, including biological medicinal products.	Whether the preparation falls within a medicinal product category governed by Union rules.	The applicable Union medicinal product pathway must be distinguished from national regimes for biomedical cell products, transplantation, and medical procedures.
Japan	Pharmaceuticals and Medical Devices Agency (PMDA) reviews regenerative medical products. Ministry of Health, Labour and Welfare (MHLW) grants marketing approval and oversees the separate framework for the provision of regenerative medicine, which includes review by certified committees [21,22,23,42].	Pharmaceuticals and Medical Devices Act and Act on the Safety of Regenerative Medicine.	Product characteristics, processing, intended use, and the distinction between a marketed product and the provision of regenerative medicine.	Marketing authorization of regenerative medical products and oversight of regenerative medicine provided by medical institutions follow separate frameworks.
China	National Medical Products Administration (NMPA) regulates cell products developed as medicines. National Health Commission (NHC) and relevant health authorities oversee biomedical technology research and clinical application within the separate institutional framework.	Medicinal product regulation and a separate framework for biomedical technologies in medical institutions.	Product characteristics and the distinction between development as a medicinal product and institution-based clinical research or application.	Medicinal product authorization must be distinguished from research filing and approval for subsequent clinical application under the biomedical technology framework. Trials conducted to develop medicines or medical devices remain subject to the corresponding legislation.
South Korea	Ministry of Food and Drug Safety (MFDS) regulates advanced biological products. Ministry of Health and Welfare (MOHW) oversees designated regenerative medicine institutions. Research oversight also involves the statutory review committee and, for specified high-risk research, MFDS approval.	Act on the Safety of and Support for Advanced Regenerative Medicine and Advanced Biological Products.	Distinction between regenerative medical interventions and biological medicinal products, degree of processing, and preservation of biological properties.	Institutional regenerative medicine and medicinal product authorization are regulated separately. Specified minimally manipulated preparations or procedures may fall outside the relevant statutory definitions.
Singapore	Health Sciences Authority (HSA) regulates CTGTP notification, registration, supply, and applicable clinical trials. Ministry of Health (MOH) oversees Class 1 CTGTP research under the Human Biomedical Research Act and specified excluded materials under healthcare services legislation.	Health Products Act and Health Products (Cell, Tissue and Gene Therapy Products) Regulations 2021.	Class 1 requires minimal manipulation, homologous use, and no combination or use in conjunction with therapeutic products or medical devices. Other CTGTPs are Class 2.	Class 1 products require an accepted notification before supply, whereas Class 2 products require registration. Specified exclusions, including qualifying use within the same surgical procedure, are assessed separately [15,33,34].
Saudi Arabia	Saudi Food and Drug Authority (SFDA) provides ATMP classification and regulates clinical trials involving these products.	Framework for advanced therapy medicinal products.	Degree of manipulation, intended function, mechanism of action, and incorporation of additional components.	Classification determines whether the preparation is assessed as a cell-based, tissue-engineered, gene therapy, or combined advanced therapy medicinal product and informs the applicable regulatory requirements.
United Arab Emirates	Emirates Drug Establishment (EDE) performs federal medical product classification and registration within its remit. Dubai Health Authority (DHA) and Department of Health Abu Dhabi (DoH) oversee healthcare facilities and services within their respective jurisdictions.	Federal medical product requirements and standards issued by individual emirates for stem cells and regenerative medicine.	Type of material, processing, intended clinical application, and requirements applicable to the product and treating facility.	Product requirements and authorization of clinical services must both be considered. Requirements issued by one emirate should not be assumed to apply throughout the federation.

**Figure 1:**
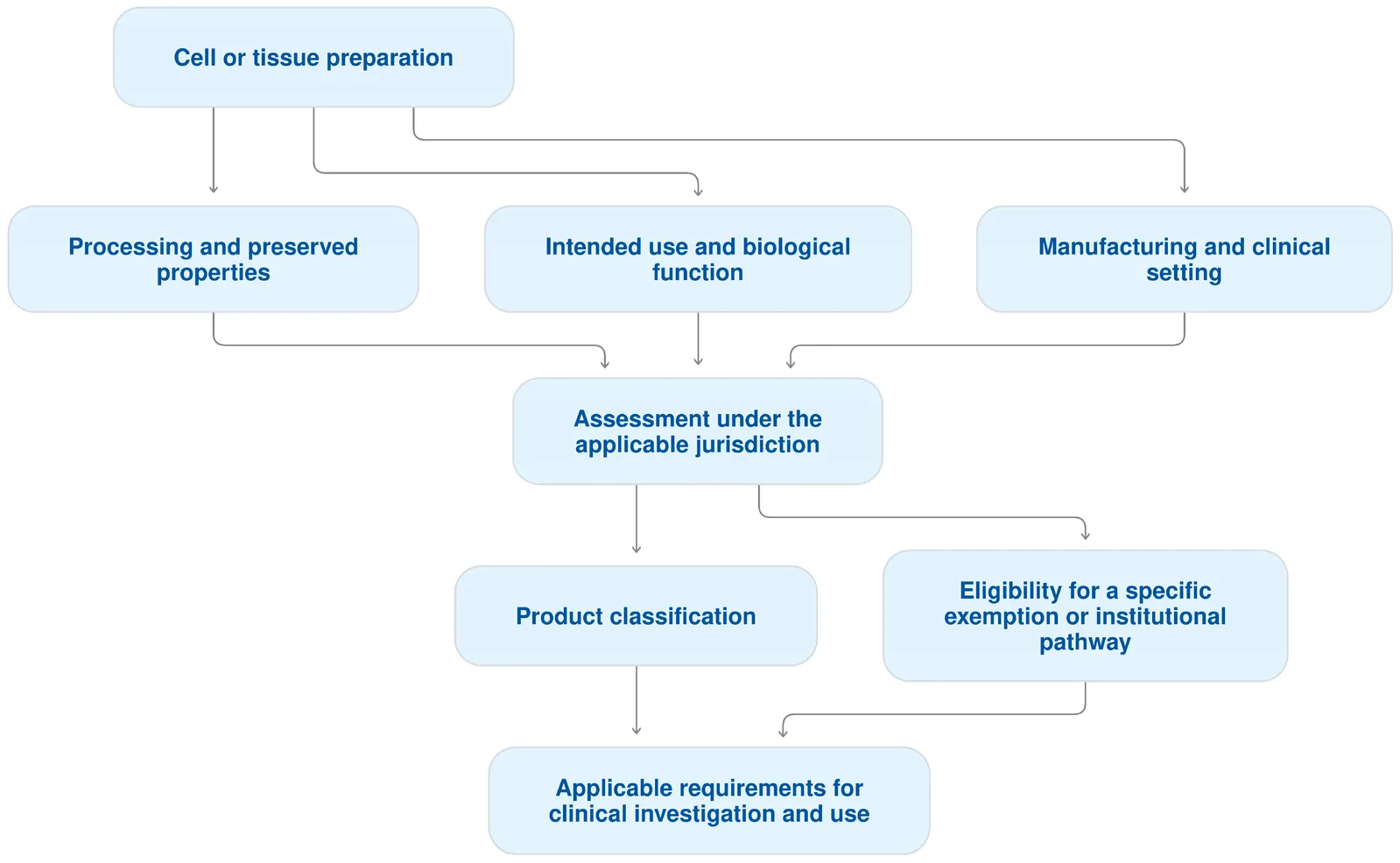
Relationship between product characteristics and regulatory assessment of cell and tissue preparations. Processing, preserved properties, intended use, and the manufacturing and clinical setting inform assessment under the applicable jurisdiction. Product classification and eligibility for a specific exemption or institutional pathway determine the relevant requirements.

Figure 1 summarizes how product characteristics inform regulatory classification and the assessment of applicable requirements for clinical investigation and use.

The comparison shows that similar product characteristics are assessed within different legal frameworks. Processing and intended function are central to several classification systems, while the distinction between a medicinal product and an intervention performed within a medical institution introduces additional requirements. These differences affect how borderline preparations are assessed. The following section examines their implications for bone marrow aspirate concentrate, adipose tissue derivatives, amniotic products, and cartilage-derived products.

## 4. Legal Assessment of Products with Contested Legal Status Across Different Countries

### 4.1. Product-Specific Regulatory Assessment

Differences between regulatory systems are most clearly revealed in the assessment of specific products whose legal status cannot be reduced unambiguously to tissue transplantation, a medical procedure, or medicinal cell therapy. These products include bone marrow aspirate concentrate, the stromal vascular fraction of adipose tissue, microfragmented adipose tissue, nanofat, amniotic products, and products derived from cartilage cells. Their legal status depends on the source material, degree of processing, preservation of tissue structure, intended use, conditions of application, and presumed mechanism of action (Table 2) [1,2,3,4].

Bone marrow aspirate concentrate is not a uniform preparation. Aspiration and concentration methods, centrifugation protocols, processing devices, additives, and intended clinical use should be specified because they inform product characterization and regulatory assessment [43,44]. Under 21 CFR 1271.3(d)(4), the exclusion from the HCT/P definition concerns minimally manipulated bone marrow for homologous use with only the permitted additions, subject to the stated safety condition. Whether a particular BMAC preparation meets these conditions requires individual assessment [1,2].

In the European Union, bone marrow aspirate concentrate is assessed with regard to the criteria of substantial manipulation and preservation of the original function of cells or tissues. If processing is limited to procurement and concentration of the material without alteration of relevant biological properties, and the use corresponds to its original function, such a product may fall outside the category of advanced therapy medicinal products. However, the final legal assessment depends on the claimed intended use, presumed mechanism of action, and the rules applicable in the specific Member State of the European Union [3,4].

For BMAC in the Russian Federation and the EAEU, uncertainty concerns the application of existing product definitions and authorization pathways to a particular preparation, rather than an absence of regulation [5,6,13,19,20]. After the 2023–2024 reform, the absence of culture expansion is not in itself a sufficient basis for excluding a product from the scope of legislation on biomedical cell products. The central issue is which legal regime should apply to such a product: transplantation material, a medical procedure, a biomedical cell product, an individualized biomedical cell product, or a high-technology medicinal product [5,6,19].

The stromal vascular fraction of adipose tissue is a more complex object of legal assessment. For application of the FDA HCT/P framework, adipose tissue is considered structural because its adipocytes and connective tissue provide cushioning and support [1]. This regulatory characterization does not deny its metabolic, endocrine, or other physiological functions. During enzymatic isolation of the stromal vascular fraction, the extracellular matrix is disrupted, and the cellular fraction is separated from the original tissue structure. Therefore, in the FDA system, enzymatically obtained stromal vascular fraction is generally regarded as a product that falls outside the scope of minimal manipulation of structural tissue [1]. The correspondence between the claimed use and the original function of adipose tissue is also relevant. If an adipose tissue-derived product is positioned as a treatment for osteoarthritis, neurodegenerative diseases, autoimmune conditions, or other disorders through anti-inflammatory, immunomodulatory, or regenerative activity, such use does not correspond to the main structural function of adipose tissue [1]. Here, SVF denotes a heterogeneous adipose-derived cellular fraction, with the isolation method specified as enzymatic or mechanical. The term does not imply a purified MSC population. Mechanically processed adipose preparations that retain tissue fragments and extracellular matrix are described separately as tissue preparations, including microfragmented adipose tissue. They are not treated as equivalent to an isolated cellular SVF preparation solely because they contain stromal vascular cells. Processing method, residual matrix, cellular composition, and viability therefore require separate reporting [45,46,47,48,49,50].

Microfragmented adipose tissue occupies an intermediate position between a tissue graft and a cell product. Unlike enzymatically isolated stromal vascular fraction, it is usually obtained mechanically, with partial preservation of the tissue structure, extracellular matrix components, and elements of the vascular-stromal niche [49,50]. For this reason, microfragmented adipose tissue is often described as less extensively processed than enzymatically isolated SVF [51,52]. In scientific publications, such products are described as minimally processed autologous adipose tissue derivatives used in orthopedics, reconstructive surgery, wound care, and other fields [43,52]. However, mechanical processing alone does not prove that a product meets the criteria for minimal manipulation. If processing substantially alters the structural properties of adipose tissue or the product is not used for its original function, a more stringent legal qualification may be required [1].

“Nanofat” denotes a heterogeneous group of mechanically emulsified adipose preparations rather than a single standardized product. Differences in emulsification, filtration, and subsequent processing can produce preparations with different cellular and extracellular-matrix characteristics [49,53,54,55]. From the perspective of legal assessment, this requires answering two questions. First, it is necessary to determine the extent to which mechanical processing has altered the original structural properties of adipose tissue. Second, it is necessary to establish whether the product is claimed as a material for soft tissue volume restoration or as a biologically active cellular fraction with regenerative and immunomodulatory activity. When nanofat is positioned primarily as a biologically active stromal cell-containing product rather than as a material for soft tissue volume restoration, a more stringent regulatory qualification becomes more likely [1,56,57].

Amniotic products demonstrate that legal classification depends on both processing method and intended use. The amniotic membrane performs barrier and protective functions. Therefore, its use as a covering or protective membrane may be regarded as close to the original function of the tissue. By contrast, the use of amniotic products to stimulate wound healing, reduce inflammation, suppress scarring, or regenerate bone tissue is regarded in the FDA guidance as nonhomologous, because such effects do not belong to the basic functions of the amniotic membrane [1]. Therefore, amniotic products cannot be assessed solely by the degree of processing. Their legal status depends on the product form, tissue preservation, presence or absence of a cellular component, claimed use, and presumed mechanism of action. This is particularly relevant for injectable, lyophilized, and combined amniotic products, whose claimed effects often extend beyond the barrier function [1,58,59].

Cartilage tissue-derived products are located at a different regulatory boundary. Transfer or mechanical processing of cartilage tissue may remain within the scope of a tissue procedure if the structural function of the material is preserved. However, cultured autologous chondrocytes, particularly when used with a matrix or membrane, no longer fall within minimally manipulated products. In the United States, MACI, which consists of autologous cultured chondrocytes on a porcine collagen membrane, has been approved by the FDA for the repair of full-thickness cartilage defects of the knee in adults [60,61]. In the European Union, MACI was classified as an advanced therapy medicinal product and, more specifically, as a tissue-engineered product because it is based on cultured autologous cells and a tissue construct [3,62].

This example shows that cell culture expansion, incorporation of cells into a matrix, and the formation of a standardized cell-tissue product require assessment within the framework of medicinal product regulation.

**Table 2:** Processing characteristics and principal regulatory considerations for selected borderline cell and tissue preparations.

Product	Processing	US Classification Considerations	EU Classification Considerations	Russia/EAEU Classification Considerations	Key Determinant of Classification
Bone marrow aspirate concentrate	Aspiration and concentration, with protocol-dependent centrifugation, devices, and additives	Assess eligibility for the specific bone marrow exclusion in 21 CFR 1271.3(d)(4). The designation BMAC alone does not establish eligibility [1,2]	Assess manipulation, intended function, and the applicable medicinal-product definition [3,17]	Distinguish transplantation material, procedural use, biomedical cell products, and medicinal products under the applicable definitions [5,6,13,19,20]	Processing, composition, permitted additions, and intended use
Isolated adipose stromal vascular fraction	Enzymatic or mechanical cellular isolation, with the method and residual matrix specified	Cell isolation that removes relevant structural components generally exceeds minimal manipulation. Intended use requires separate assessment [1,2]	Assess substantial manipulation or a different essential function together with the relevant ATMP definition [3,17]	Assess cellular composition, manufacturing, intended action, and the applicable product category [5,13,19,20]	Disruption of tissue architecture, cell isolation, and intended action
Microfragmented adipose tissue	Mechanical fragmentation with protocol-dependent retention of tissue and extracellular matrix	Mechanical processing alone does not establish minimal manipulation. Preserved characteristics and intended use require assessment [1,2]	Assess changes in relevant properties and intended function under the applicable product definition [3,17]	Assess the resulting tissue preparation and conditions of manufacture and use [5,6,13,19,20]	Preserved tissue properties, extent of fragmentation, and intended use
Nanofat preparations	Mechanical emulsification with variable filtration and further processing	Assess changes in structural characteristics and the claimed use [1,2]	Assess the resulting preparation against manipulation, function, and medicinal-product criteria [3,17]	Assess the characterized preparation rather than relying on the clinical label “nanofat” [5,13,19,20]	Protocol-dependent composition, retained matrix, and intended action
Amniotic products	Processing varies by product, including preservation, dehydration, micronization, or combination	Assess relevant tissue properties and homologous use. Membrane covering and claims of regenerative activity require different functional assessments [1,2,58,59]	Assess processing, composition, intended action, and the relevant tissue or medicinal-product framework [3,17,63]	Assess processing, cellular composition, intended use, and the applicable legal category [5,13,19,20]	Barrier function, cellular content, dosage form, and therapeutic claims
Cartilage tissue and chondrocyte-derived products	Tissue transfer or processing, or cell isolation and expansion with possible scaffold incorporation	Distinguish tissue preparations from defined cultured chondrocyte products, including the approved MACI product [1,2,60]	Assess tissue preparations separately from cultured cell constructs that meet tissue-engineered product criteria [3,62]	Distinguish transplantation materials and procedural use from biomedical cell and medicinal products [5,13,19,20]	Cell expansion, preserved function, scaffold incorporation, and intended use

The table identifies assessment considerations rather than definitive classifications of all preparations within each row. Processing characteristics are not standardized by the product names. Eligibility for an exemption or institutional pathway requires a separate assessment. ATMP, advanced therapy medicinal product. EAEU, Eurasian Economic Union.

### 4.2. Mesenchymal Stromal Cell-Based Interventions and MSC-Containing Preparations

The regulatory relevance of MSCs depends on the composition of the material administered to the patient. Adipose-derived stromal vascular fraction (SVF) is a heterogeneous suspension that contains stromal cells alongside endothelial, perivascular, and immune cell populations. Microfragmented adipose tissue retains fragments of extracellular matrix and elements of native tissue architecture, while the cellular composition of nanofat varies with the processing protocol [45,49,51,53,55]. These preparations should be distinguished from isolated or culture-expanded MSCs. In particular, the total nucleated cell count in SVF is not an MSC dose, and the presence of stromal cells does not establish their contribution to a clinical effect [9,10].

The distinction is consequential when evidence from one type of preparation is used to support another. Results obtained with culture-expanded MSCs cannot be assumed to apply to SVF or microfragmented adipose tissue, even when all three are described as stromal cell-based interventions. Their composition, processing, and proposed mechanisms of action must be evaluated separately.

In the United States, assessment of adipose-derived products includes whether processing alters the structural characteristics of adipose tissue and whether the intended use is homologous. Describing SVF or nanofat as acting through MSC-mediated anti-inflammatory or immunomodulatory effects is relevant to the assessment of intended use, but such a claim does not establish the identity of the active cell population or clinical efficacy [1,2]. In the European Union, substantial manipulation or use for a different essential function may bring a cell or tissue product within the advanced therapy medicinal product framework. A culture-expanded MSC preparation and mechanically processed adipose tissue therefore require separate assessments [3,4,11]. In the Russian Federation, the absence of culture expansion alone is insufficient to determine whether a preparation falls outside the framework for biomedical cell products. Its composition, manufacturing setting, and intended use must be considered under the applicable rules [5,6,19].

Product composition, a proposed mechanism of action, and evidence demonstrating that mechanism should be reported separately. When therapeutic activity is attributed specifically to MSCs, the administered preparation requires appropriate phenotypic characterization and functional assessment relevant to the proposed activity. Neither total nucleated cell count nor marker expression alone establishes MSC dose or therapeutic potency. Clinical improvement alone also does not identify the responsible cell population or mechanism. Reports should therefore distinguish measured product attributes from mechanistic hypotheses and supporting experimental evidence [9,10,11,64,65].

Clinical development of defined MSC products provides examples of how product characterization and indication-specific evidence inform regulatory decisions. In December 2024, the FDA approved remestemcel-L-rknd (Ryoncil), an allogeneic bone marrow-derived MSC product, for steroid-refractory acute graft-versus-host disease in pediatric patients aged 2 months and older. The supporting study included 54 treated pediatric patients and assessed response at day 28. This approval established a treatment option for a defined population, although the absence of a concurrent control group limits comparative interpretation of the clinical results [66,67].

Darvadstrocel illustrates the importance of confirming an initial efficacy finding. The ADMIRE-CD trial reported higher combined remission with expanded allogeneic adipose-derived cells than with placebo in adults with complex perianal fistulas associated with Crohn’s disease [68]. The larger ADMIRE-CD II trial did not confirm superiority for its primary endpoint [69]. The European Commission subsequently withdrew the marketing authorization for Alofisel in December 2024 at the holder’s request. EMA reported that the available evidence no longer established sufficient clinical benefit, while the confirmatory trial identified no new safety signals [70].

Evidence on borderline preparations also requires controlled evaluation. In the MILES trial, BMAC, SVF, and umbilical cord tissue-derived MSCs were assessed separately against corticosteroid injection for knee osteoarthritis. None demonstrated superiority for the primary pain outcomes at 12 months [71].

Defined MSC products offer manufacturing options that differ from preparations obtained during a clinical procedure. Culture expansion permits production of specified cell quantities, and cryopreservation can support storage and subsequent administration. These features are used in the manufacture of Ryoncil [67]. Their implementation requires control of product identity, viability, sterility, and biological activity. Donor characteristics, culture conditions, and processing changes may affect product attributes, while surface marker expression alone does not establish potency for a proposed indication [9,11].

The selected studies and their quantitative results are summarized in Table 3. Together, these examples illustrate the need to connect clinical claims to the administered product, manufacturing process, target population, and measured outcome. Evidence from a defined MSC product cannot establish the efficacy of an uncultured heterogeneous preparation without additional supporting data.

These results require interpretation within their original study designs. A response proportion from a single-arm study does not estimate the benefit over an alternative treatment. Likewise, improvement from baseline in a randomized trial does not establish superiority unless the between-group comparison supports that conclusion. The different findings of the two darvadstrocel trials also show why a positive initial result requires confirmation. Differences in indications, products, comparators, endpoints, and safety definitions preclude a pooled estimate across the examples presented here [66,67,68,69,70,71].

## 5. Practical Recommendations for Researchers, Clinicians, and Healthcare Institutions

**Figure 2:**
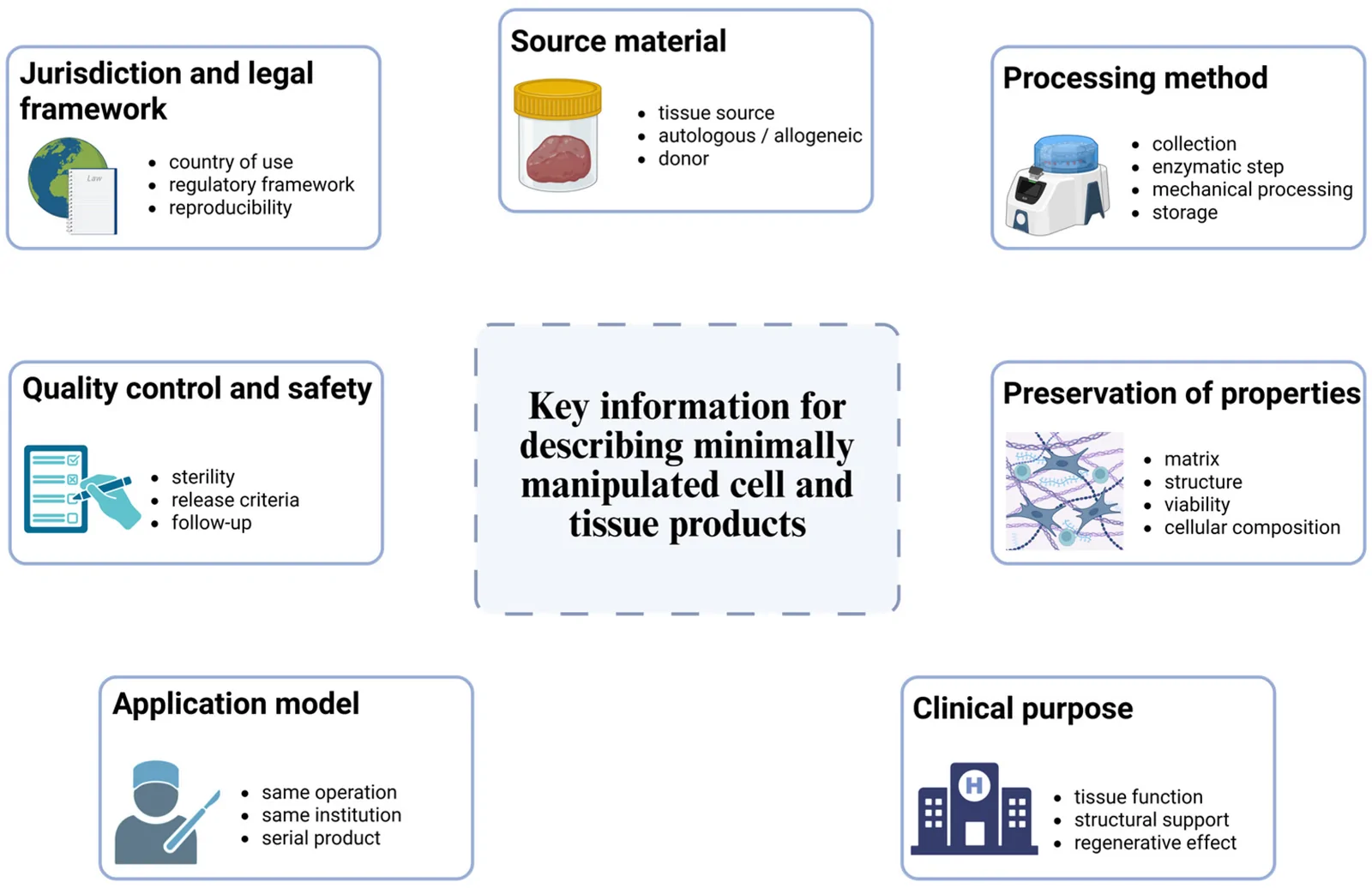
Key elements required for the standardized description of minimally processed cell and tissue products in scientific publications and clinical protocols.

The comparative analysis supports a minimum reporting framework for minimally processed cell and tissue products in scientific publications and clinical protocols (Figure 2). Incomplete product descriptions may limit legal assessment, comparison between studies, and reproduction of the technology in another jurisdiction [9,10,64,65].

First, the anatomical source of the tissue and the nature of the material, whether autologous or allogeneic, should be specified. For allogeneic material, information on the donor, donor selection procedure, and infectious disease testing should also be provided, since these data determine the applicable regulatory regime regardless of the extent of subsequent processing [2,19,63].

A sequential description of the processing method is also required. This should include the method of material procurement, the presence or absence of an enzymatic step with specification of the reagents and their concentrations, mechanical processing procedures, auxiliary substances, and storage and transport conditions [1,2,3,4].

For structural tissues, the degree of preservation of the extracellular matrix, spatial organization, and mechanical characteristics should be reported. For cellular and nonstructural products, cell viability, phenotypic composition, and biological activity relevant to the intended use should be described [1,2,11]. When an MSC-mediated effect is claimed for a heterogeneous preparation, authors should state how the stromal cell population was identified and quantified rather than using the total nucleated cell count as a surrogate for MSC dose [9,10].

The precise formulation of the clinical intended use of the product and its relationship to the original function of the donor tissue are also relevant. Use of a product for soft tissue volume restoration, structural support, or barrier protection corresponds to one model of legal assessment, whereas claims of regenerative, anti-inflammatory, immunomodulatory, or systemic effects generally lead to a more stringent qualification [1,2,3,4]. The relationship between the claimed function and the original properties of the tissue is one of the main criteria for distinguishing a tissue procedure from a cellular medicinal product.

Publications should also specify the mode of use: whether the product is used intraoperatively within the same surgical procedure, manufactured in the same medical organization in which it is used, or produced serially. These features determine the applicability of special exemptions: the US same surgical procedure exception, the European hospital exemption for advanced therapy medicinal products, and the Russian regime for individualized biomedical cell products [2,3,6,17].

Finally, regardless of the applicable legal regime, product release criteria, methods for sterility and viability control, and the procedure for post-application patient follow-up should be described. A less stringent regulatory pathway does not eliminate the risks of contamination, variability in cellular composition, or unpredictable biological activity [72,73,74]. Since the same product may have different legal status in different countries, publications should directly specify the country of application and, where possible, the legal regime under which the product was used. This allows readers to relate the reported experience to the regulation in their own country and assess the feasibility of reproducing the technology in another legal system [4,14,15,33].

Compliance with these requirements reduces terminological uncertainty, limits the improper use of the category of minimal processing as a basis for exemption from regulatory oversight, and creates conditions for appropriate comparison of study results obtained in different legal systems.

## 6. Limitations

This review is subject to several limitations. The level of detail differs across jurisdictions, owing to variation in the availability of primary regulatory materials in English and in the extent to which enforcement practice is publicly accessible. The regulatory framework governing cell and tissue products is developing rapidly. Consequently, certain provisions discussed in this review may be revised before publication. Although selected enforcement actions and judicial decisions were examined, we did not systematically survey enforcement practice or clinical use in every jurisdiction considered. The clinical examples were selected for their relevance to the regulatory discussion and do not represent an exhaustive assessment of MSC-based interventions. Their results cannot establish comparative effectiveness across products or indications, and no pooled efficacy or safety estimate was calculated.

## 7. Conclusions

Minimally manipulated cell and tissue products are not regulated as a uniform legal category across the jurisdictions analyzed. Their legal status is determined by the source material, processing method, preservation of structural and biological properties, intended use, manufacturing conditions, and clinical setting. Autologous origin, absence of culture expansion, or intraoperative use alone does not represent sufficient grounds for applying a simplified regulatory regime [1,2,3,19]. Mechanical processing alone likewise does not establish compliance with a minimal-manipulation criterion [1,2].

The same product may receive different legal qualifications when the intended use, processing method, or jurisdiction changes. This is particularly relevant for adipose tissue-derived products, amniotic products, bone marrow aspirate concentrate, and cartilage-derived products, whose classification often depends on the relationship between preserved tissue function and the claimed clinical effect. The development of US case law concerning stromal vascular fraction and FDA warning letters on umbilical cord and amniotic products shows that enforcement practice can substantially influence the interpretation of regulatory criteria [58,75,76].

Differences between regulatory systems affect the requirements for evidence, quality control, manufacturing documentation, clinical evaluation, informed consent, adverse event reporting, and follow-up. Clinical data generated within one legal system cannot be transferred directly to another without considering differences in product qualification, manufacturing conditions, and post-application surveillance. Current harmonization initiatives within ICH, APACRM, and the reform of European pharmaceutical legislation mainly address documentation, quality control, clinical studies, and post-authorization surveillance. They do not remove differences in the criteria used for legal classification [77,78,79].

Regulatory assessment of minimally manipulated cell and tissue products therefore requires both product characterization and analysis of current enforcement practice. Scientific publications and clinical protocols should provide a standardized description of the source material, processing method, preserved properties, intended use, manufacturing conditions, model of clinical application, and jurisdiction. These elements are necessary for reproducibility, comparison of clinical data, and assessment of whether the reported technology can be applied safely and lawfully in another regulatory system.

These distinctions are particularly important when stromal cell-containing tissues are compared with isolated or culture-expanded MSCs. Evidence obtained with one preparation should not be generalized to another without documenting its cellular composition, processing method, and proposed therapeutic action.

## Figures and Tables

**Table 3:** Selected clinical studies relevant to the regulatory assessment of MSC products and heterogeneous cell preparations.

Study and Intervention	Design and Population	Main Efficacy Result	Safety Findings	Interpretation
MSB-GVHD001. Remestemcel-L-rknd, allogeneic bone marrow-derived MSCs [66,67]	Prospective, multicenter, single-arm study. Fifty-four pediatric patients with steroid-refractory acute graft-versus-host disease received treatment.	Overall response at day 28 occurred in 38/54 patients, reported as 70% with a 95% CI of 56.4% to 82.0%. Median response duration was 54 days.	The prescribing information reports serious adverse reactions in 35/54 patients (65%).	Supported FDA approval for a specified pediatric population. The absence of a concurrent control group limits estimation of comparative benefit and excess treatment-associated risk.
ADMIRE-CD. Darvadstrocel, expanded allogeneic adipose-derived cells [68]	Phase 3 randomized controlled trial. Adults with complex perianal fistulas associated with Crohn’s disease. Darvadstrocel *n* = 107, placebo *n* = 105.	Combined remission at week 24 in the ITT population occurred in 53/107 versus 36/105 patients, reported as 50% versus 34%. Difference 15.2 percentage points, 97.5% CI 0.2 to 30.3, *p* = 0.024.	Treatment-related adverse events occurred in 18/103 versus 30/103 patients in the safety populations.	Initial evidence of benefit in a defined indication. Efficacy and safety analyses used different denominators.
ADMIRE-CD II. Darvadstrocel [69,70]	Phase 3 randomized placebo-controlled trial. Darvadstrocel *n* = 283, placebo *n* = 285. Both groups underwent fistula curettage and closure of internal openings.	Combined remission at week 24 occurred in 138/283 (48.8%) versus 132/285 (46.3%). Estimated difference 2.4 percentage points, 95% CI −5.8 to 10.6, *p* = 0.571.	Treatment-emergent adverse events occurred in 203/278 (73.0%) versus 201/274 (73.4%). No new safety signals were identified.	The primary efficacy endpoint was not met. These findings informed reassessment of the clinical benefit of Alofisel in the EU.
MILES. BMAC, SVF, and umbilical cord tissue-derived MSCs compared with corticosteroid injection [71]	Multicenter randomized trial in knee osteoarthritis. A total of 480 participants were randomized, with 440 included in the primary endpoint analyses.	Mean VAS pain changes at 12 months were −24.3 for BMAC, −19.4 for SVF, −20.1 for MSCs, and −20.9 for corticosteroid injection. None of the cellular interventions was superior for the VAS or KOOS pain co-primary endpoints.	No procedure-related serious adverse events were reported during the study.	Improvement from baseline did not establish superiority over the active comparator. Absence of superiority does not demonstrate equivalence between the preparations.

CI, confidence interval. ITT, intention-to-treat. BMAC, bone marrow aspirate concentrate. SVF, stromal vascular fraction. MSC, mesenchymal stromal cell. VAS, visual analogue scale. KOOS, Knee injury and Osteoarthritis Outcome Score. Combined remission in the darvadstrocel trials required closure of all treated external fistula openings and absence of collections larger than 2 cm on MRI. Percentages and between-group estimates are presented as reported by the original sources. Safety definitions and analysis populations differed among studies, preventing direct comparison of adverse-event frequencies across products and indications.

## Data Availability

The original contributions presented in this study are included in the article. Further inquiries can be directed to the corresponding author.
